# Gravity induces asymmetric Ca^2+^ spikes in the root cap in the early stage of gravitropism

**DOI:** 10.1080/15592324.2021.2025325

**Published:** 2022-01-13

**Authors:** Ruoxin Zhao, Zonghao Liu, Ziwei Li, Shi Xu, Xianyong Sheng

**Affiliations:** College of Life Sciences, Capital Normal University, Beijing, China

**Keywords:** Root, gravitropism, vertical stage microscope, Ca^2+^ spikes, the lateral root cap

## Abstract

Gravitropism is an important strategy for the adaptation of plants to the changing environment. Previous reports indicated that Ca^2+^ participated in plant gravity response. However, present information on the functions of Ca^2+^ in plant gravitropism was obtained mainly on coleoptiles, hypocotyls, and petioles, little is known about the dynamic changes of Ca^2+^ during root gravitropism. In the present study, the transgenic *Arabidopsis thaliana* R-GECO1 was placed horizontally and subsequently vertically on a refitted Leica SP8 laser scanning confocal microscopy with a vertical stage. Real-time observations indicated that gravistimulation induced not only an increase in the Ca^2+^ concentration, but also an accelerated occurrence of Ca^2+^ sparks in the root cap, especially in the lower side of the lateral root cap, indicating a strong tie between Ca^2+^ dynamics and gravistimulation during the early stage of root gravity response.

Plants can sense gravity stimulation, and adjust the growth direction of their organs, such as roots and stems, to obtain sufficient sunlight, water, and minerals necessary for growth and development.^1–3^ Even though plant gravitropism has been discovered for more than 100 years and statocytes, such as the root cap, has long been considered to play an indispensable role in plant gravitropic response, little is known about the molecular and cellular mechanisms behind which plants translate physical force into biochemical signals.^2–8^ In particular, the initial biological signals induced by gravity stimulation in statocytes remain a fascinating and perplexing problem.

As an intracellular second messenger, Ca^2+^ has long been regarded as the initial biological signal.^9–14^ In fact, Ca^2+^ signals induced by gravitational stimulation have been repeatedly reported in the past few decades. For example, using Ca^2+^ fluorescent indicators or transgenic Ca^2+^-reporter systems, increases in Ca^2+^ concentration in response to gravistimulation were already demonstrated in multiple plant species.^15–17^ Besides, pharmacological treatments with extra cellular Ca^2+^ chelatorsor, Ca^2+^-channel blockers, as well as inhibitors of calmodulin or Ca^2+^/calmodulin dependent protein kinases, inevitably led to altered root gravitropism.^10,18,19^ All these data indicated that Ca^2+^ might play a vital role in plant gravitropic response.

Unfortunately, previous studies were carried out mainly on coleoptiles, hypocotyls, and/or petioles, and only a few data were obtained from roots. Besides, due to limited imaging technologies, the temporal and spatial resolution of previous researches was difficult to define the specific cell types where Ca^2+^ dynamics happened, let alone to clarify which phase of gravitropism was affected by Ca^2+^ dynamics. Especially, plant bending growth results from gravity-induced asymmetric cell elongation,^2,5,6,8^ conventional cell live-imaging technologies, including laser scanning confocal microscopy, are incompetent for synchronous visualization and quantification of the information between the upper and lower sides of statocytes after gravity stimulation. Hence, real-time observations on intracellular changes of signaling molecules, such as Ca^2+^ and pH, with a vertical-stage microscope are very promising.^20–23^

As noninvasive Ca^2+^ reporters, genetically encoded indicators (GECIs) are appropriate tools to study spatiotemporal information of Ca^2+^ dynamics in living cells.^24–26^ R-GECOs are red emitting reporters generated by replacing the cpGFP with cpmApple. Among these reporters, R-GECO1 is an intensity-based Ca^2+^ reporter capable of monitoring Ca^2+^ dynamics in plants.^25,26^ To further understand gravity-induced dynamic changes of Ca^2+^ in the root, seeds of transgenic *Arabidopsis thaliana* R-GECO1 were cultured in 1/2 Murashige & Skoog (MS) with 1% sucrose for 5 days. Seedlings were positioned on the glass bottom of a Petri dish, covered with fresh culture medium containing 1/2 MS, and placed vertically in an artificial climate box for 30 min. Then, the Petri dish was placed on a Leica SP8 laser scanning confocal microscopy refitted with a vertical stage and a 40× oil objective (NA 1.3). All samples were excited with 561 nm and fluorescence emission was detected between 570 and 640 nm using a HyD detector. Laser power and channel setting were kept identical for all samples to make the results comparable. Firstly, time-series images were obtained with root grew vertically down for 15 min. Subsequently, the Petri dish was placed horizontally and time-series images were obtained for additional 60 min. The results indicated that bright fluorescence signals were easily detected in the whole root cap (Figure 1). Surprisingly, it was occasionally observed in both the columella and the lateral root cap cells that the mean fluorescence intensity suddenly increased by about 30–70% within 12.06 s ± 6.31 s (n = 22), and then quickly returned to original baseline levels within 21.0 ± 7.18 s (n = 22), suggesting the existence of Ca^2+^ sparks in these cells (Figure 1, Supplemental video 1).

**Figure 1.**
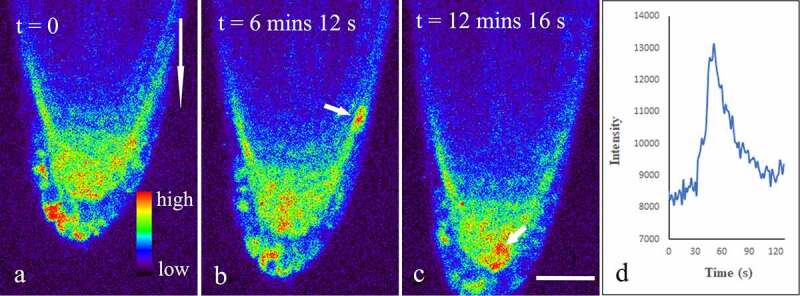
Dynamic changes of Ca^2 +^ in vertically cultured Arabidopsis roots. Seedlings of five-day transgenic *A. thaliana* R-GECO1 were whole-mounted in a Petri dish with fresh 1/2 MS. A total of more than 15 roots were placed vertically on a Leica SP8 laser scanning confocal microscope refitted with a vertical stage. Time-lapse images about 15 min (about 600 frames at 1.48 s per frame) were captured. The short arrows show Ca^2 +^ Sparks in the lateral root cap (b), and the columella cells (c), respectively. The long arrow shows the direction of gravity. Figure D shows the dynamic changes in mean fluorescence intensity of a lateral root cap cell during a Ca^2 +^ Sparks. Bar: 50 μm.

Starch-filled amyloplasts within the columella cells sedimenting in the direction of gravity were believed to trigger the generation of a signal that causes asymmetric growth.^27–29^ In the present study, real-time observations revealed that within 1 min after the roots were placed horizontally on the stage, a rapid increase in the concentration of Ca^2+^ in the root cap was detected (Figure 2). In fact, quantitative analysis using Leica LAS X showed that the mean Ca^2+^ fluorescence intensity of the horizontally placed columnar cells was 10286 ± 1721 (n = 13), which was significantly higher than that of vertically placed roots (9102 ± 1603, n = 13, *t*-test, *p* < 0.05). Similarly, the mean fluorescence intensity of the cells in the lower side of the lateral root cap was significantly increased from 9603 ± 1684 to 11467 ± 2079 (n = 13, *p* < 0.05) by gravity stimulation. However, no marked change in the mean fluorescence intensity was detected in the upper side of the lateral root cap cells (9967 ± 2349, n = 13, *p* > 0.05). These data are consistent with previous reports that gravistimulation induced a specific Ca^2+^ increase in the horizontally oriented coleoptiles, hypocotyl, and petiole.^11,13,14,16,17^ Thus, we might here further speculate that the increase in the concentration of Ca^2+^ was a universal phenomenon existing in various plant organs responding to gravistimulation.

**Figure 2.**
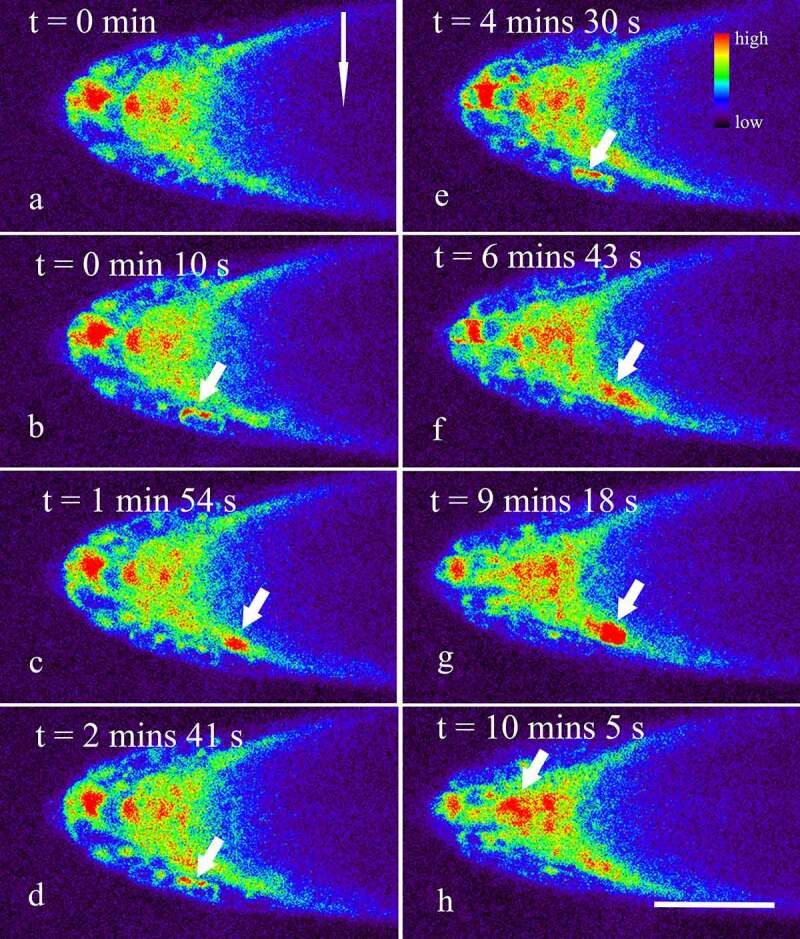
Dynamic changes of Ca^2+^ in horizontally placed Arabidopsis roots during the next 15 min after the onset of the gravistimulation. Seedlings of five-day transgenic *A. thaliana* R-GECO1 were whole-mounted in a Petri dish with fresh 1/2 MS. A total of more than 15 roots were placed horizontally on a Leica SP8 laser scanning confocal microscope refitted with a vertical stage. Time-lapse images about 15 min (about 600 frames at 1.48 s per frame) were captured. The short arrows show Ca^2+^ Sparks occurring mainly in the lower side of the lateral root cap (a-g), and occasionally occurring in the columella cells (h). The long arrow shows the direction of gravity. Bar: 50 μm.

Besides, though gravity stimulation did not significantly affect the amplitude and duration of Ca^2+^ sparks in the root cap (*p* > 0.05), an accelerated occurrence of Ca^2+^ sparks in the lower side of the lateral root cap were indeed observed, especially within 15 min after the onset of the gravistimulation (Figure 2, Supplemental video 2). In fact, the frequency of Ca^2+^ sparks in vertically placed lateral root caps was about 1.58 ± 0.23 per 10 min (n = 13), while the data in the lower and upper sides of the lateral root caps were about 6.28 ± 2.76 and 1.39 ± 0.89 per 10 min (n = 13), respectively, indicating that gravity stimulation resulted in an asymmetrical increase in the frequencies of Ca^2+^ sparks in the lateral root caps (*p* < 0.001). With the lapse of time, the frequency of Ca^2+^ sparks in the lower sides of the lateral root caps reduced gradually (Figure 3a–f, Supplemental video 3). Finally, no significant difference in the frequencies of Ca^2+^ sparks between the upper and lower sides was observed when the root was horizontally placed for about 45–60 min (Figure 3g–h, Supplemental video 4).

**Figure 3.**
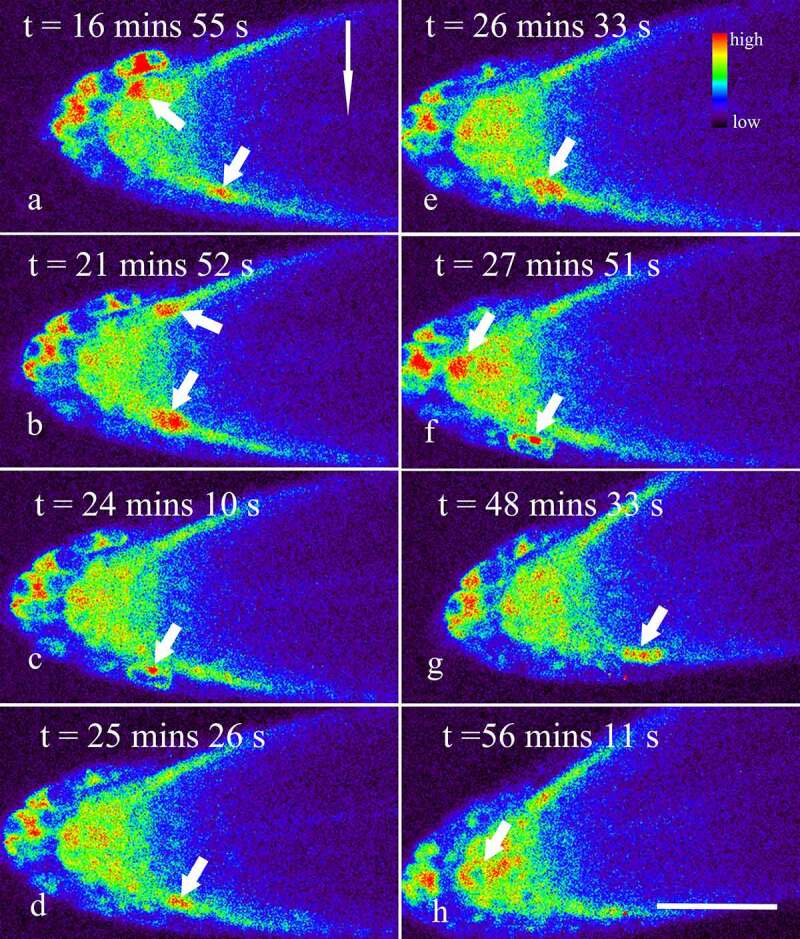
Dynamic changes of Ca^2+^ in horizontally placed Arabidopsis roots during 15–60 min after the onset of the gravistimulation. Seedlings of R-GECO1 were prepared and placed horizontally on a vertical stage laser scanning confocal microscope, as mentioned above. Dynamic changes of Ca^2+^ in the root cap during 15–60 min after the onset of the gravistimulation were captured at 1.48 s per frame. The short arrows show Ca^2+^ Sparks occurring in both the lower and upper sides of the lateral root cap (a-g), and occasionally in the columella cells (f, h). The long arrow shows the direction of gravity. Bar: 50 μm.

On the other hand, though the mean Ca^2+^ fluorescence intensity of the horizontally placed columnar cells was significantly higher than that of vertically placed roots, no significant difference in the frequency of Ca^2+^ sparks between vertically and horizontally placed columella cells was observed (n = 13, *p* > 0.05). This phenomenon might be due to the fact that the columella cells are located inside the root cap,^30^ and Ca^2+^ dynamics, especially in the inner layers of the columella cells, are relatively difficult to be detected. Given that the presentation time for Arabidopsis roots is approx. 0.4 min,^31^ and that it would take approx. 0.5 min for us to rotate the vertically positioned sample by 90 degrees, another possibility that cannot be excluded is that, due to the limitations of the technique used, our data might have missed earlier signal changes occurring in the root columella cells.

Transcellular calcium currents were regarded as the earliest changes directed by gravity in Spores of the fern *Ceratopteris richardii*.^32^ Gravistimulation-induced Ca^2+^ waves moved from the root tip toward the elongation zone on the lower side of the root were also observed in roots of *A. thaliana*.^15^ In the present study, each Ca^2+^ spark occurred strictly within a single cell. No obvious signal to transmit from one cell to neighboring cells was observed (Figures 1–3, Supplemental videos 1–4), indicating that there was no significant temporal and spatial correlation between the cells where Ca^2+^ sparks occured.

Since root gravitropic response began quickly after the onset of a gravity stimulus, the initial phase of gravitropism cannot rely on newly synthesized proteins and, therefore must be non-genomic.^2,6,33^ In the present, an increase in Ca^2+^ concentration, as well as the Ca^2+^ sparks, can be detected within 1 min after gravistimulation, suggesting that Ca^2+^ dynamics were indeed induced by gravistimulation (Figure 2, Supplemental video 2). However, we still do not know how Ca^2+^ plays a role during root gravitropism. Especially, it was still impossible to confirm whether Ca^2+^ was the initial signal or the transmission signal of the root in response to a gravity stimulus. Perhaps, gravity stimulation somehow induced the changes in the Ca^2+^ concentration, as well as the frequencies of Ca^2+^ sparks, which in consequence triggered the relocalization of PINs, such as PIN3 and PIN7, and an auxin asymmetry essential for gravitropic bending between the upper and lower side of the root.^34–36^ On the other hand, auxin can somehow induce Ca^2+^ signals,^17,37–39^ which in turn promotes differential apoplastic pH changes at both sides of the gravistimulated root,^15^ resulting in root curvature soon after the onset of the gravistimulation. Anyhow, Ca^2+^ dynamics in the root cap might be one of the most important signaling in the early phase of the tropic response.

## Supplementary Material

Supplemental Material
